# Validation of Hematological Markers in Early Onset Neonatal Sepsis

**DOI:** 10.7759/cureus.26446

**Published:** 2022-06-30

**Authors:** Deepshikha Rana, Himadri Hazarika, Aditi Agarwal, Richa Gupta, Mrinalini Kotru

**Affiliations:** 1 Pathology, University College of Medical Sciences, New Delhi, IND; 2 Pathology, Dr. Ram Manohar Lohia Hospital, Delhi, IND; 3 Pathology, University College of Medical Sciences, Delhi, IND

**Keywords:** biomarker, sepsis, neonates, lymphocyte, neutrophil

## Abstract

Background

Neonatal sepsis is considered a ubiquitous worldwide cause of mortality and morbidity in newborn infants. The incidence is 10-50 per 1000 live births. Neutrophil to lymphocyte ratio (NLR) is an easily accessible and cost-effective hematological marker for prompt diagnosis of neonatal sepsis.

Aim and objectives

The purpose of this study was to analyze the clinical significance of NLR in neonates clinically diagnosed with sepsis and its impact on the management.

Methods

This retrospective study was conducted on 265 neonates diagnosed with sepsis and compared with 341 healthy controls. The statistical analysis was performed by using the Student's t-test to compare the variables.

Result

Median NLR levels were significantly higher in patients than in controls. NLR had a modest power of predicting neonatal sepsis, as suggested by an area under a curve of 0.569.

Conclusion

NLR is an important predictor of neonatal sepsis. There is a significant modest positive correlation between NLR and sepsis.

## Introduction

Sepsis can be defined as a life-threatening organ dysfunction caused by an abnormally regulated host response to infection [1]. It is usually a fatal complication of infection, particularly in underdeveloped and developing countries, where it represents a major cause of maternal and neonatal morbidity and mortality [2]. Many studies have revealed that failure of early recognition and prompt management can lead to septic shock, multiple organ failure, and death. Although numerous clinical biomarkers have been extensively explored for this purpose, only a few have been currently applied in clinical practice. This has led to a continued search for a desirable marker that may facilitate early intervention as well as predict the prognosis of sepsis [3].

The commonly used biomarkers to identify sepsis include lactate, pro-inflammatory cytokines, and chemokines such as C-reactive protein and calcitonin. Recently, anti-inflammatory cytokines and alterations in cell surface markers have also been studied. But a majority of these biomarkers are highly-priced, not readily available, and/or not validated [4]. Complete blood count (CBC), on the other hand, is a primary investigation in sepsis screening and is available at almost all centers [5]. This makes the neutrophil to lymphocyte ratio (NLR) a readily available parameter that can be computed from CBC. With this background, we conducted this study and sought to evaluate the significance of NLR in neonatal sepsis.

## Materials and methods

This study was conducted retrospectively in the Department of Pathology of a tertiary care center in the University College of Medical Sciences, catering to the population of North and North-East Delhi, including the adjoining districts of Uttar Pradesh. A total of 265 neonates were included in this study that spanned the year 2021. The clinical diagnosis of sepsis was made in an outpatient department (OPD) by doctors using various clinical signs and symptoms, keeping into account the weeks of gestation, birth weight, and clinical characteristics like aversion to feeding, somnolence, irritability, lethargy, low perfusion, and temperature irregularities. The diagnosis requires the presence of two or more systemic inflammatory response syndrome (SIRS) criteria in case of strong clinical suspicion. It is defined as (1) temperature >38.5°C or <36°C; (2) mean heart rate >2 SD above normal for age in the absence of external stimuli, or unexplained persistent elevation for children less than one years old, or mean heart rate <10th percentile for age, or unexplained persistent depression over a 0.5hr time period; (3) mean respiratory rate of >2 SD above normal for age or in the presence of mechanical ventilation; and (4) abnormal leukocyte count or >10% immature neutrophils [6]. Various parameters, such as hemoglobin, red blood cell (RBC) count, packed cell volume, mean corpuscular volume (MCV), mean corpuscular hemoglobin (MCH), mean corpuscular hemoglobin concentration (MCHC), total leucocyte count, differential leukocyte count, and platelet count were obtained from an automated analyzer (Beckman Coulter, Brea, California). The clinical data was also taken into account; any newborn with any associated conditions such as hematological disease, major congenital malformation, and congenital cyanotic heart disease was excluded from the study.

We collected complete laboratory data of gestational age-matched 341 neonates who were healthy and had no clinical symptoms suggestive of sepsis as the control group.

Statistical analysis

All the continuous variables were statistically expressed as mean and standard deviation (SD). A comparison of mean ± SD of total leucocyte count (TLC), absolute neutrophil count (ANC), absolute lymphocyte count (ALC), and NLR between the sepsis and control groups were carried out by application of an independent t-test. The diagnostic accuracy of NLR for neonatal sepsis was assessed by the receiver operating characteristic (ROC) curve, and sensitivity, specificity, positive predictive value (PPV), and negative predictive value (NPV) were calculated. P-values of <0.05 were considered statistically significant. For the analysis of the above data, we used the statistical software SPSS version 20.0 (IBM Inc., Armonk, New York).

## Results

A total of 265 septics and 341 non-septic neonates were included in this study. Comparison of TLC, ANC, and NLR between the two groups showed higher values in the cases with p-values of <0.001, 0.001, and 0.026, respectively, all of which were statistically significant. However, ALC was lower in cases than in the controls, with a statistically significant p-value of 0.004 (Table 1)

**Table 1:** Independent t-test to compare the values of cases and controls RBC - red blood cell, MCV - mean corpuscular volume, MCH - mean corpuscular hemoglobin, MCHC - mean corpuscular hemoglobin concentration, TLC - total leucocyte count, ANC - absolute neutrophil count, ALC - absolute lymphocyte count, NLR - neutrophil to lymphocyte ratio

Hematologic parameters	Cases (n=256)	Controls (n=341)	t-test	p-value
	Mean ± SD	Mean ± SD		
Hemoglobin	16.32±3.24	11.02±1.64	24.015	<0.001
RBC count	4.65±0.92	4.22±0.93	5.623	<0.001
Hematocrit	49.37±9.81	34.88±4.29	22.095	<0.001
MCV	106.37±8.68	84.27±9.51	29.146	<0.001
MCH	35.62±4.87	26.72±3.56	25.773	<0.001
MCHC	33.4±3	31.53±1.64	9.045	<0.001
Platelet count	1.88±0.99	2.48±2.09	-4.257	<0.001
TLC	14.09±12.3	10.28±3.09	4.837	<0.001
ANC	70.66±7.59	68.43±8.57	3.361	0.001
ALC	20.37±6.7	22.04±7.41	-2.855	0.004
NLR	4.05±2.09	3.67±1.99	2.234	0.026

An analysis of the ROC curve was done to assess the sensitivity of NLR to predict sepsis. The area under the curve (AUC), optimal cut-off value, sensitivity, specificity, PPV, NPV, and diagnostic accuracy are presented in Table 2.

**Table 2:** Sensitivity, specificity, positive predictive value, negative predictive value, and diagnostic accuracy of the neutrophil to lymphocyte ratio

Parameter	Area under curve	Sensitivity	Specificity	Positive predictive value	Negative predictive value	Diagnostic accuracy	Sensitivity
NLR	0.569	3.4	56.60%	52.5%	47.20%	61.70%	54.27%

**Figure 1:**
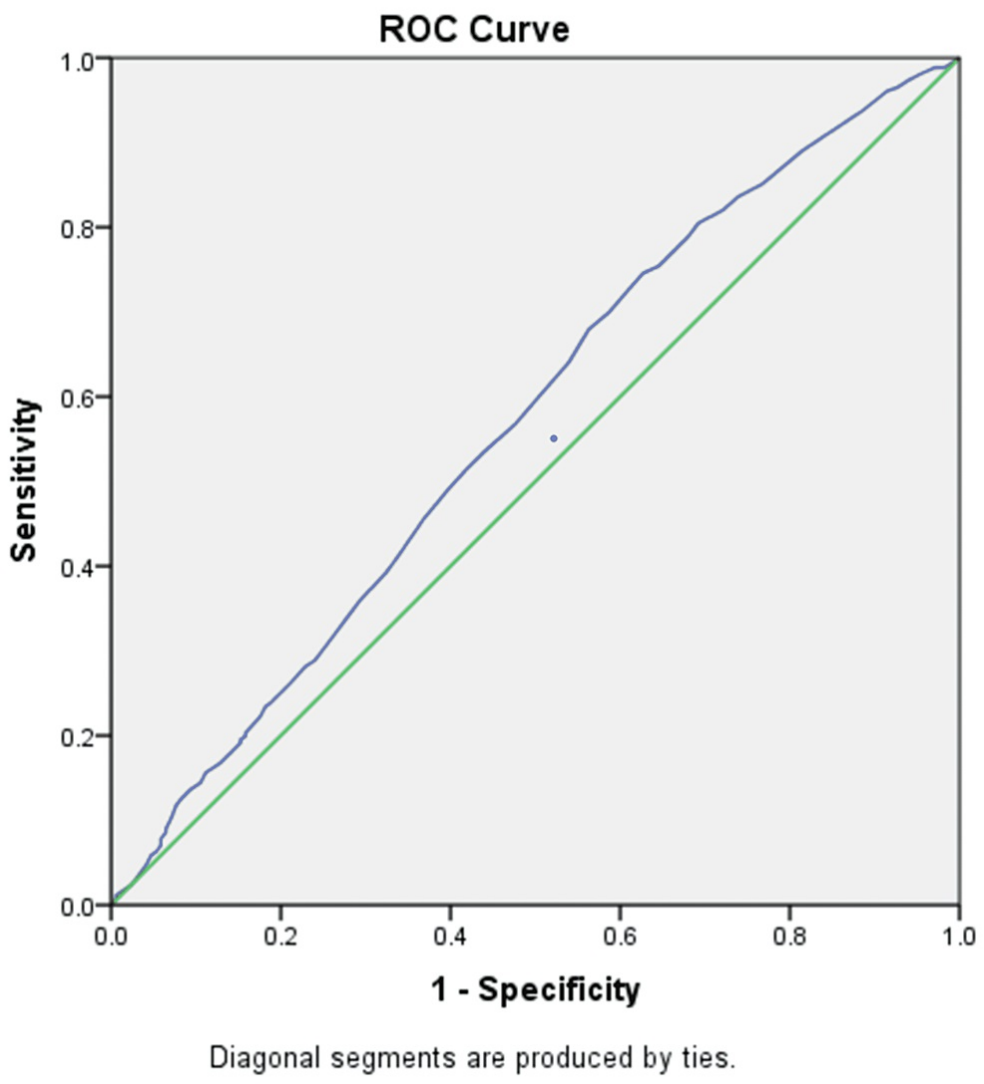
ROC curve showing specificity and sensitivity of NLR along the x-axis and y-axis, respectively, with an area under curve of 0.569 which indicates a good predictability for sepsis NLR - neutrophil to lymphocyte ratio, ROC - receiver operating characteristic

NLR had an AUC of 0.569, indicating good (not excellent) predictability. The NLR at a cut-off of 3.4 had a sensitivity of 56.6% and specificity of 52.5%. The test had a positive predictive value of 47.2% and a negative predictive value of 61.7% (Figure 1).

## Discussion

Neonatal sepsis is a major issue to deal with worldwide and contributes to significant morbidity and mortality, especially in developing countries with an incidence of 10-50 per 1000 live births [7]. India has the highest incidence of sepsis (17,000/1,00,000 live births), with the case fatality rate of neonatal sepsis ranging between 25% to 65% [8]. Its diagnosis is usually difficult and challenging. For the diagnosis of neonatal sepsis, blood culture is considered the gold standard. However, it is time-consuming, which could delay intervention and lead to a wide distribution of pathogenic organisms [9]. Therefore, identifying a rapid, sensitive and specific biomarker is critical.

NLR cogitates the association between the circulating numbers of neutrophils and lymphocytes in the whole body. Neutrophils, as cells of first-line defense, play a crucial role in innate immunity providing rapid sensing and elimination of pathogens. Lymphocytes, on the other hand, are the essential cell type of adaptive immunity, which provide a wider and more cell reserve for recognition of antigens [10]. Delayed apoptosis of neutrophils, as well as the accelerated release of immature neutrophils, together with assist in the enormous elevation of circulating neutrophils in septic patients, augmenting neutrophil-mediated killing. This is a response of an innate immune system that causes phenomenal tissue damage. In addition to this response, immunosuppressive cytokine IL-10 is enormously produced by the neutrophils during sepsis to promote infection. Apart from neutrophils, lymphocytes also play a pivotal role in continuing appropriate inflammatory responses. In the thymus and spleen, the increased apoptosis of lymphocytes can result in severe immunosuppression, multiple organ failure, and even death which can be linked to a prolonged, detrimental inflammatory state. Henceforth, the NLR epitomizes the balance between innate and adaptive immunity [11]. Furthermore, the NLR can be considered a convenient, easily accessible biomarker that can be analyzed by using the whole blood cell count. The current study showed a significantly higher mean NLR in cases than in controls. This was in concordance with studies conducted by Panda et al. [5], Mira et al. [9], and Li et al. [12]. The mean NLR in septic neonates in the present study was 4.05±2.09, which almost corresponded to the mean NLR value of 3.88±1.78 by Panda et al. [5]. Significantly higher NLR in suspected septic neonates compared to healthy neonates were also noted in several studies. The NLR of the present study had an area under the curve of 0.569, indicating good predictability.

Triumph of neonatal sepsis lies in premature intervention, for which we require a complete understanding of early features of neonatal sepsis and extensive interpretation of laboratory investigations. There have been numerous studies regarding the usage of C-reactive protein (CRP) and blood culture for the diagnosis. However, these studies have a more waiting period, and blood culture has a low positive rate [13, 14]. It should also be noted that these modalities cannot be replaced and will still remain the gold standard for the diagnosis of neonatal sepsis. However, in this study, we hope to highlight the potential value of NLR in prompt identification of cases and thus expedite the management on day 1 of admission, thereby preventing life-threatening consequences.

The limitations of the study, however, were that it was a single-center study, and we did not follow up with the blood culture report. This was because we wanted to highlight the importance of NLR as an independent marker of neonatal sepsis, owing to the time-consuming cost of blood culture.

## Conclusions

One of the major causes of ICU admissions is neonatal sepsis, and it is also linked to very high hospital costs along with worse outcomes in neonates. Our primary aim is to reduce these outcomes for both the patient and the hospital. The present study highlights the value of NLR as a preliminary marker in clinically suspected neonatal sepsis cases, particularly in developing countries. It is a relatively easy and cost-effective test.

## References

[1] Singer M, Deutschman CS, Seymour CW (2016). The third international consensus definitions for sepsis and septic shock (sepsis-3). JAMA.

[2] (2020). Sepsis. https://www.who.int/news-room/fact-sheets/detail/sepsis.

[3] Liu X, Shen Y, Wang H, Ge Q, Fei A, Pan S (2016). Prognostic significance of neutrophil-to-lymphocyte ratio in patients with sepsis: a prospective observational study. Mediators Inflamm.

[4] George AA, Thomas TP, Gaffor A (2020). The role of neutrophil/lymphocyte ratio in predicting the severity of sepsis in a tertiary care hospital in South India: a retrospective study. Int J Res Med Sci.

[5] Panda SK, Nayak MK, Rath S, Das P (2021). The utility of the neutrophil-lymphocyte ratio as an early diagnostic marker in neonatal sepsis. Cureus.

[6] Goldstein B, Giroir B, Randolph A (2005). International pediatric sepsis consensus conference: definitions for sepsis and organ dysfunction in pediatrics. Pediatr Crit Care Med.

[7] Sumitro KR, Utomo MT, Widodo AD (2021). Neutrophil-to-lymphocyte ratio as an alternative marker of neonatal sepsis in developing countries. Oman Med J.

[8] Murthy S, Godinho MA, Guddattu V, Lewis LE, Nair NS (2019). Risk factors of neonatal sepsis in India: a systematic review and meta-analysis. PLoS One.

[9] Mira SM, Elkhaleegy HM, Elbakry EMT, Abd-Elraheem SI (2021). Neutrophil and platelet to lymphocyte ratio for detecting early-onset neonatal sepsis. IJMA.

[10] Liu S, Wang X, She F, Zhang W, Liu H, Zhao X (2021). Effects of neutrophil-to-lymphocyte ratio combined with interleukin-6 in predicting 28-day mortality in patients with sepsis. Front Immunol.

[11] Ye W, Chen X, Huang Y (2020). The association between neutrophil-to-lymphocyte count ratio and mortality in septic patients: a retrospective analysis of the MIMIC-III database. J Thorac Dis.

[12] Li T, Dong G, Zhang M (2020). Association of neutrophil-lymphocyte ratio and the presence of neonatal sepsis. J Immunol Res.

[13] Scheer CS, Fuchs C, Gründling M (2019). Impact of antibiotic administration on blood culture positivity at the beginning of sepsis: a prospective clinical cohort study. Clin Microbiol Infect.

[14] Lamy B, Dargère S, Arendrup MC, Parienti JJ, Tattevin P (2016). How to optimize the use of blood cultures for the diagnosis of bloodstream infections? A state-of-the art. Front Microbiol.

